# Editorial: New technologies improve maternal and newborn safety

**DOI:** 10.3389/fmedt.2024.1372358

**Published:** 2024-05-30

**Authors:** Jieyun Bai, Yaosheng Lu, Huishu Liu, Fang He, Xiaohui Guo

**Affiliations:** ^1^ Guangdong Provincial Key Laboratory of Traditional Chinese Medicine Information Technology, Jinan University, Guangzhou, China; ^2^ Auckland Bioengineering Institute, University of Auckland, Auckland, New Zealand; ^3^ Guangzhou Women and Children’s Medical Center, Guangzhou Medical University, Guangzhou, China; ^4^ Department of Obstetrics and Gynecology, Third Affiliated Hospital of Guangzhou Medical University, Guangzhou, China; ^5^ Department of Obstetrics, Shenzhen People’s Hospital, Shenzhen, China

**Keywords:** digital twin, intrapartum ultrasound, electronic fetal monitoring, uterine electromyography, cardiotocography, artificial intelligence, deep learning

**Editorial on the Research Topic** New technologies improve maternal and newborn safety

## 1 Introduction

Daily, it's reported that 800 women and 6,700 newborns lose their lives during or shortly after childbirth. Furthermore, approximately 5,400 babies are stillborn each day, with 40% of these losses occurring during the birthing process (1). A significant portion of these stillbirths, maternal deaths, neonatal fatalities, and injuries are preventable through the provision of safe, respectful, and quality care during pregnancy, childbirth, and the early days of a newborn's life. The World Health Organization (WHO) encourages healthcare facility administrators, policymakers, and healthcare providers worldwide to adopt five principal goals for World Patient Safety Day 2021 (2). These goals are directed at improving the safety of mothers and newborns at critical healthcare moments, particularly during childbirth (2). The goals encompass: “(1) Reducing unnecessary and harmful interventions for women and newborns during childbirth; (2) Strengthening the support and skills of healthcare workers to provide safe care for mothers and infants; (3) Promoting respectful care to ensure a positive birth experience; (4) Improving the safe use of medications and blood transfusions during childbirth; and (5) Methodically recording and analyzing safety incidents related to childbirth” (2).

There's a profound interest in pioneering innovations that enhance the safety of mothers and newborns, particularly in addressing the dangers posed by inadequate maternal and neonatal care during pregnancy, childbirth, and the initial postnatal period (3, 4). However, significant challenges hinder the efficacy and affordability of existing interventions. As such, this synopsis aggregates the recent breakthroughs (5) and methodologies (6–11), delves into potential impact on improving maternal and infant safety, and reflects on how these advancements may guide future academic research.

## 2 Intrapartum ultrasound in assessing labor dynamics

Approximately half of the world's stillbirths, as well as maternal and neonatal deaths, stem from complications during labor, delivery, and the immediate postnatal phase, especially prevalent in regions with limited resources (12). While these fatalities are largely avoidable through prompt interventions like cesarean sections, there are apprehensions surrounding both their underutilization and overutilization (13). Historically, the primary role of obstetric ultrasound has been in prenatal screenings for fetal anomalies (14). Yet, its utility in monitoring labor progression is emerging, bolstered by an increasing corpus of evidence attesting to its capability to objectively evaluate labor dynamics (15). The advent of true intrapartum ultrasound, an innovative facet of this technology, is gaining ground. This approach has illuminated the complex physiological mechanisms of labor, offering detailed insights into the phases of childbirth and potentially forecasting the outcomes of instrumental vaginal births. Nonetheless, the technique's complexity and susceptibility to inaccuracies, particularly when operated by obstetricians without specialized ultrasound training, cannot be overlooked. In this context, the integration of Artificial Intelligence (AI) could be revolutionary. AI has the potential to streamline and refine this process, improving the accuracy of measurements and diminishing the dependency on the individual clinician's expertise (16–19).

The Grand Challenge on Pubic Symphysis-Fetal Head Segmentation (PSFHS) from Transperineal Ultrasound Images (https://doi.org/10.5281/zenodo.7861699), a segment of the 26th International Conference on Medical Image Computing and Computer-Assisted Intervention (MICCAI), marks a significant stride in this arena (https://ps-fh-aop-2023.grand-challenge.org/) (20, 21). This challenge drew over 100 teams to develop AI algorithms specifically for obstetric ultrasound imaging. The goal was not limited to analyzing images; it also encompassed the assurance that these AI solutions conform to clinical standards while assessing biometric parameters accurately (6, 22–25). The triumph of the MICCAI-PSFHS Challenge underscores the evolution of advanced intrapartum ultrasound technology, highlighting not just technological progress but also the potential of AI models to predict the most suitable delivery methods. For instance, we launched the Intrapartum Ultrasound Grand Challenge (IUGC) (https://zenodo.org/records/10979813) as part of MICCAI 2024 (https://codalab.lisn.upsaclay.fr/competitions/18413) (26). This challenge calls for the development of automatic, user friendly systems for fetal biometrics, aiming to minimize intra and inter observer variability and enhance the reliability of measurements. Such advancements could revolutionize labor management, blending the precision of technology with the nuances of human care.

## 3 Biosignal-based methods for fetal-maternal monitoring

Continuous fetal heart rate (FHR) monitoring via cardiotocography (CTG) stands as the primary technique for assessing fetal well-being during labor, simultaneously tracking FHR and uterine contractions (UC) (27). This dual monitoring allows for real-time analysis of these critical parameters. The extraction of FHR and UC data predominantly relies on either invasive or non-invasive methods, with the latter being more commonly used. Specifically, non-invasive methods like Doppler ultrasound and the tocodynamometer involve attaching two external transducers to the mother's abdomen. Despite their widespread use, these signals often encounter interference from fetal or maternal movements and may diminish in quality as maternal body mass index increases. This limitation in CTG data reliability poses a substantial challenge in meeting the performance criteria necessary for its extensive clinical deployment (7, 9, 28–32). This challenge underscores the urgent need for innovative monitoring techniques such as the non-invasive fetal electrocardiogram (33) and electrohysterogram (34) to improve the fundamental data quality vital for developing automated systems.

In response to this need, the biennial Workshop on Signal Processing and Monitoring in Labor (SPaM) serves as a collaborative platform, promoting a range of interdisciplinary research approaches and innovations. The SPaM Workshop (https://www.wrh.ox.ac.uk/research/spam-in-labour) aims to cultivate a truly interdisciplinary arena and create a shared language among clinicians, physiologists, and signal processing specialists (35). The development of novel data-driven methods for CTG analysis during labor necessitates comprehensive datasets that encapsulate uncommon clinical situations. Currently, only a limited number of public CTG datasets are available: (1) The Czech Technical University and University Hospital in Brno (CTU-UHB) dataset (36), which includes 552 CTG recordings with unprocessed FHR and UC signals, and (2) the Lille dataset, which contains 156 CTG recordings from the obstetric clinic at Saint Vincent de Paul Hospital (Lille, France) (37). Jinan University also provides two datasets under a data sharing agreement: one with 784 signals for signal categorization (29), and another with 331 signals for automated feature extraction from signals (7, 32).

The past decade has seen an influx of machine learning and deep learning approaches in the medical field, prompting numerous studies focusing on the analysis of CTG signals (38–45). Modern systems demonstrate impressive efficacy in detecting fetal hypoxia in retrospective patient groups. Nevertheless, several challenges must be overcome to facilitate their integration into clinical practices. Primarily, creating and disseminating comprehensive, open, and anonymized multicentric databases of perinatal and CTG data from labor is crucial to enhance system precision (31). Furthermore, these systems should provide comprehensible metrics along with risk assessments for fetal hypoxia to build trust and acceptance among medical professionals. Finally, it's vital to establish and adhere to universal evaluation standards for these systems using retrospective patient groups and to validate their clinical utility.

## 4 Biophysics-based computer modelling

The successful progression of labor is closely associated with changes in cervical compliance, particularly evident through cervical shortening. The proper timing of these uterine changes is crucial, as deviations can lead to significant clinical consequences. Notably, premature uterine activation, often accompanied by early cervical shortening, can result in preterm birth, affecting an estimated 15 million infants worldwide each year, as reported by the WHO (46). These early births significantly heighten the risk of neonatal death (constituting more than half of all neonatal deaths) and various long-term health issues. The relatively limited understanding of the physiology behind uterine activation constrains our ability to enhance clinical interventions for severe pregnancy complications such as preterm birth and uterine dystocia. Recognizing the potential of multi-scale computational modeling of the uterus is gaining momentum. This approach aims to integrate diverse pieces of information into a unified, predictive, and testable model of uterine behavior, thereby informing the creation of new diagnostic and treatment strategies for these pressing clinical challenges (47).

While uterine models offer an alternative to *in vivo* experiments on animal and human subjects through simulations, authentic data from these subjects are crucial for developing a uterine model that provides clinically relevant insights (48–50). Therefore, noninvasive methods of data collection are incredibly valuable. Pioneering work by researchers at Washington University School of Medicine in St. Louis has led to the development of innovative imaging technology that enables real-time, three-dimensional visualizations of the intensity and spread of uterine contractions across the entire surface of the uterus during labor (51). This technology, an extension of imaging techniques previously used for the heart, provides a noninvasive, intricately detailed view of uterine contractions, surpassing the capabilities of current tools that only detect the presence of contractions (52, 53). Although advancements in data recording technology have streamlined the process of gathering authentic clinical data, a significant portion of research still proceeds without experimental data. Even when experimental data is incorporated into research, the collected information may not be sufficient or suitable for both the development and validation of models.

## 5 Digital twin in fetal-maternal health

Digital twins (DTs) in healthcare represent sophisticated virtual models of patients, created by integrating individual patient data, wider population statistics, and real-time updates related to patient-specific and environmental variables (54). These DTs for precision health are complex virtual constructs designed to emulate the anatomy, context, and behavior of human bodies or healthcare systems, including their interconnections. They are regularly updated with information from their real-life counterparts and are characterized by their predictive functionality. The validity of a DT can be verified, making it an invaluable tool for decision-making, providing critical insights to inform the delivery of health and wellness care (8). At the heart of the digital twin concept is the dynamic, two-way communication between the virtual and physical realms. This continuous flow of data from the human health system to the computational model ensures the digital twin remains in lockstep with the human health system. Such a close alignment significantly improves the capacity to identify risk factors based on current or expected behaviors and/or adverse events. Although DTs are a relatively new concept in healthcare compared to other industries, they have demonstrated potential across various sectors of precision medicine (55, 56). Applications include managing chronic diseases like asthma and diabetes, tailored cancer treatments, personalized cardiovascular system models (57–60), and predictive simulations for treatment responses in infectious diseases. However, integrating DTs into healthcare presents several challenges and obstacles that must be addressed. Overcoming these challenges is imperative for DTs to fulfill their promise as a cutting-edge framework for individual health management and healthcare services.

In today's advancing landscape, sophisticated technologies such as medical imaging, data analytics, and AI are reshaping prenatal care for pregnant women and fetuses (61–64). These technologies significantly enhance the accuracy and efficacy of healthcare services, signifying a profound shift towards more individualized and predictive healthcare approaches. As these technologies continue to mature and merge with the digital twin concept, they are poised to unlock unparalleled capabilities in the monitoring, diagnosis, and treatment of health conditions, potentially transforming the realm of maternal and fetal medicine.

## 6 Conclusions

The research marks a significant stride forward in enhancing maternal and newborn safety, setting the stage for notable advancements in diagnosis and treatment. The promise of these developments lies not just in the individual technologies, but in the synergy of multidisciplinary research, the seamless integration of cutting-edge technologies, and the tailoring of care to individual needs. This holistic approach is pivotal in revolutionizing fetal-maternal health, promising to elevate the quality of life for countless patients globally. The path forward is one of collaboration and relentless innovation, leading to a future where fetal-maternal monitoring transcends its current boundaries to become more precise, efficacious, and universally accessible, thereby transforming the landscape of maternal and newborn healthcare.

## References

[1] Ronsmans C Graham WJ. Maternal mortality: who, when, where, and why. Lancet. (2006) 368(9542):1189–200. 10.1016/S0140-6736(06)69380-X 17011946

[2] WHO’s world patient safety day goals 2021 promote safe maternal and newborn practices. Saudi Med J. (2021) 42(11):1259–60. 34732564 PMC9149736

[3] Liu J Wang C Yan R Lu Y Bai J Wang H Machine learning-based prediction of postpartum hemorrhage after vaginal delivery: combining bleeding high risk factors and uterine contraction curve. Arch Gynecol Obstet. (2022) 306(4):1015–25. 10.1007/s00404-021-06377-0 35171347

[4] Fernandez-Turienzo C Sandall J. Delivering high-quality childbirth care. Nat Med. (2024) 30(2):348–9. 10.1038/s41591-024-02812-2 38336834

[5] Vogel JP Pujar Y Vernekar SS Armari E Pingray V Althabe F Effects of the WHO labour care guide on cesarean section in India: a pragmatic, stepped-wedge, cluster-randomized pilot trial. Nat Med. (2024) 30(2):463–9. 10.1038/s41591-023-02751-4 38291297 PMC10878967

[6] Ou Z Bai J Chen Z Lu Y Wang H Long S RTSeg-Net: a lightweight network for real-time segmentation of fetal head and pubic symphysis from intrapartum ultrasound images. Comput Biol Med. (2024):108501. 10.1016/j.compbiomed.2024.108501 38703545

[7] Liu M Zeng R Xiao Y Lu Y Wu Y Long S Automated fetal heart rate analysis for baseline determination using EMAU-net. Inf Sci (Ny). (2023) 644:119281. 10.1016/j.ins.2023.119281

[8] Calcaterra V Pagani V Zuccotti G. Maternal and fetal health in the digital twin era. Front Pediatr. (2023) 11:1251427. 10.3389/fped.2023.1251427 37900683 PMC10601630

[9] Liu M Lu Y Long S Bai J Lian W. An attention-based CNN-BiLSTM hybrid neural network enhanced with features of discrete wavelet transformation for fetal acidosis classification. Expert Syst Appl. (2021) 186:115714. 10.1016/j.eswa.2021.115714

[10] Erickson EN Gotlieb N Pereira LM Myatt L Mosquera-Lopez C Jacobs PG. Predicting labor onset relative to the estimated date of delivery using smart ring physiological data. NPJ Digit Med. (2023) 6(1):153. 10.1038/s41746-023-00902-y 37598232 PMC10439919

[11] Coutinho-Almeida J Cardoso A Cruz-Correia R Pereira-Rodrigues P. Fast healthcare interoperability resources-based support system for predicting delivery type: model development and evaluation study. JMIR Form Res. (2024) 8:e54109. 10.2196/54109 38587885 PMC11036185

[12] Hug L You D Blencowe H Mishra A Wang Z Fix MJ Global, regional, and national estimates and trends in stillbirths from 2000 to 2019: a systematic assessment. Lancet. (2021) 398(10302):772–85. 10.1016/S0140-6736(21)01112-0 34454675 PMC8417352

[13] Miller S Abalos E Chamillard M Ciapponi A Colaci D Comandé D Beyond too little, too late and too much, too soon: a pathway towards evidence-based, respectful maternity care worldwide. Lancet. (2016) 388(10056):2176–92. 10.1016/S0140-6736(16)31472-6 27642019

[14] Fiorentino MC Villani FP Di Cosmo M Frontoni E Moccia S. A review on deep-learning algorithms for fetal ultrasound-image analysis. Med Image Anal. (2023) 83:102629. 10.1016/j.media.2022.102629 36308861

[15] Ghi T Eggebø T Lees C Kalache K Rozenberg P Youssef A ISUOG practice guidelines: intrapartum ultrasound. Ultrasound Obstet Gynecol. (2018) 52(1):128–39. 10.1002/uog.19072 29974596

[16] Ramirez Zegarra R Ghi T. Use of artificial intelligence and deep learning in fetal ultrasound imaging. Ultrasound Obstet Gynecol. (2023) 62(2):185–94. 10.1002/uog.26130 36436205

[17] Slimani S Hounka S Mahmoudi A Rehah T Laoudiyi D Saadi H Fetal biometry and amniotic fluid volume assessment end-to-end automation using deep learning. Nat Commun. (2023) 14(1):7047. 10.1038/s41467-023-42438-5 37923713 PMC10624828

[18] Rueda S Fathima S Knight CL Yaqub M Papageorghiou AT Rahmatullah B Evaluation and comparison of current fetal ultrasound image segmentation methods for biometric measurements: a grand challenge. IEEE Trans Med Imaging. (2014) 33(4):797–813. 10.1109/TMI.2013.2276943 23934664

[19] van den Heuvel TLA de Bruijn D de Korte CL Ginneken BV. Automated measurement of fetal head circumference using 2D ultrasound images. PLoS One. (2018) 13(8):e0200412. 10.1371/journal.pone.0200412 30138319 PMC6107118

[20] Lu Y Zhou M Zhi D Zhou M Jiang X Qiu R The JNU-IFM dataset for segmenting pubic symphysis-fetal head. Data Brief. (2022) 41:107904. 10.1016/j.dib.2022.107904 35198683 PMC8842023

[21] Chen G Bai J Ou Z Lu Y Wang H. PSFHS: intrapartum ultrasound image dataset for AI-based segmentation of pubic symphysis and fetal head. Sci Data. (2024) 11(1):402. 10.1038/s41597-024-03233-z 38698003 PMC11066050

[22] Chen Z Ou Z Lu Y Bai J. Direction-guided and multi-scale feature screening for fetal head–pubic symphysis segmentation and angle of progression calculation. Expert Syst Appl. (2024) 245:123096. 10.1016/j.eswa.2023.123096

[23] Lu Y Zhi D Zhou M Lai F Chen G Ou Z Multitask deep neural network for the fully automatic measurement of the angle of progression. Comput Math Methods Med. (2022) 2022:5192338. 10.1155/2022/5192338 36092792 PMC9462992

[24] Bai J Sun Z Yu S Lu Y Long S Wang H A framework for computing angle of progression from transperineal ultrasound images for evaluating fetal head descent using a novel double branch network. Front Physiol. (2022) 13:940150. 10.3389/fphys.2022.940150 36531181 PMC9755498

[25] Zhou M Wang C Lu Y Qiu R Zeng R Zhi D The segmentation effect of style transfer on fetal head ultrasound image: a study of multi-source data. Med Biol Eng Comput. (2023) 61(5):1017–31. 10.1007/s11517-022-02747-1 36645647

[26] Qiu R Zhou M Bai J Lu Y Wang H. PSFHSP-Net: an efficient lightweight network for identifying pubic symphysis-fetal head standard plane from intrapartum ultrasound images. Med Biol Eng Comput. (2024). 10.1007/s11517-024-03111-1. [Epub ahead of print]. PMC11379789 38722478

[27] INFANT Collaborative Group. Computerised interpretation of fetal heart rate during labour (INFANT): a randomised controlled trial. Lancet. (2017) 389(10080):1719–29. 10.1016/S0140-6736(17)30568-8 28341515 PMC5413601

[28] Zhong M Yi H Lai F Liu M Zeng R Kang X CTGNet: automatic analysis of fetal heart rate from cardiotocograph using artificial intelligence. Maternal-Fetal Med . (2022) 4(2). 10.1097/FM9.0000000000000147 PMC12094348 40406444

[29] Xiao Y Lu Y Liu M Zeng R Bai J. A deep feature fusion network for fetal state assessment. Front Physiol. (2022) 13:969052. 10.3389/fphys.2022.969052 36531165 PMC9748093

[30] Zeng R Lu Y Long S Wang C Bai J. Cardiotocography signal abnormality classification using time-frequency features and ensemble cost-sensitive SVM classifier. Comput Biol Med. (2021) 130:104218. 10.1016/j.compbiomed.2021.104218 33484945

[31] Bai J Pan X Lu Y Zhong M Wang H Zheng Z Comparison of fetal heart rate baseline estimation by the cardiotocograph network and clinicians: a multidatabase retrospective assessment study. Front Cardiovasc Med. (2023) 10:1059211. 10.3389/fcvm.2023.1059211 37621563 PMC10445644

[32] Liu M Zeng R Xiao Y Bai J Liu J Zheng Z Baseline/acceleration/deceleration determination of fetal heart rate signals using a novel ensemble LCResU-net. Expert Syst Appl. (2023) 218:119610. 10.1016/j.eswa.2023.119610

[33] Kahankova R Martinek R Jaros R Behbehani K Matonia A Jezewski M A review of signal processing techniques for non-invasive fetal electrocardiography. IEEE Rev Biomed Eng. (2019) 13:51–73. 10.1109/RBME.2019.2938061 31478873

[34] Jager F. An open dataset with electrohysterogram records of pregnancies ending in induced and cesarean section delivery. Sci Data. (2023) 10(1):669. 10.1038/s41597-023-02581-6 37783671 PMC10545725

[35] Georgieva A Abry P Chudácek V Djuric PM Frasch MG Kok R Computer-based intrapartum fetal monitoring and beyond: a review of the 2nd workshop on signal processing and monitoring in labor (October 2017, Oxford, UK). Acta Obstet Gynecol Scand. (2019) 98(9):1207–17. 10.1111/aogs.13639 31081113 PMC7135636

[36] Chudácek V Spilka J Burša M Janku P Hruban L Huptych M Open access intrapartum CTG database. BMC pregnancy childbirth. (2014) 14:1–12. 24418387 10.1186/1471-2393-14-16 PMC3898997

[37] Boudet S Houzé de l’Aulnoit A Demailly R Peyrodie L Beuscart R Houzé de l’Aulnoit D. Fetal heart rate baseline computation with a weighted median filter. Comput Biol Med. (2019) 114:103468. 10.1016/j.compbiomed.2019.103468 31577964

[38] Aeberhard JL Radan AP Delgado-Gonzalo R Strahm KM Sigurthorsdottir HB Schneider S Artificial intelligence and machine learning in cardiotocography: a scoping review. Eur J Obstet Gynecol Reprod Biol. (2023) 281:54–62. 10.1016/j.ejogrb.2022.12.008 36535071

[39] Ben M'Barek I Jauvion G Ceccaldi PF. Computerized cardiotocography analysis during labor—a state-of-the-art review. Acta Obstet Gynecol Scand. (2023) 102(2):130–7. 10.1111/aogs.14498 36541016 PMC9889319

[40] Chen Y Guo A Chen Q Quan B Liu G Li L Intelligent classification of antepartum cardiotocography model based on deep forest. Biomed Signal Process Control. (2021) 67:102555. 10.1016/j.bspc.2021.102555

[41] Lovers AAK Ugwumadu A Georgieva A. Cardiotocography and clinical risk factors in early term labor: a retrospective cohort study using computerized analysis with Oxford system. Front Pediatr. (2022) 10:784439. 10.3389/fped.2022.784439 35372157 PMC8966702

[42] Mendis L Palaniswami M Brownfoot F Keenan E. Computerised cardiotocography analysis for the automated detection of fetal compromise during labour: a review. Bioengineering (Basel). (2023) 10(9):1007. 10.3390/bioengineering10091007 37760109 PMC10525263

[43] Ben M’Barek I Jauvion G Vitrou J Holmström E Koskas M Ceccaldi PF. DeepCTG® 1.0: an interpretable model to detect fetal hypoxia from cardiotocography data during labor and delivery. Front Pediatr. (2023) 11:1190441. 10.3389/fped.2023.1190441 37397139 PMC10311205

[44] Cao Z Wang G Xu L Li C Hao Y Chen Q Intelligent antepartum fetal monitoring via deep learning and fusion of cardiotocographic signals and clinical data. Health Inf Sci Syst. (2023) 11(1):16. 10.1007/s13755-023-00219-w 36950107 PMC10025176

[45] Ricciardi C Improta G Amato F Cesarelli G Romano M. Classifying the type of delivery from cardiotocographic signals: a machine learning approach. Comput Methods Programs Biomed. (2020) 196:105712. 10.1016/j.cmpb.2020.105712 32877811

[46] Chawanpaiboon S Vogel JP Moller AB Lumbiganon P Petzold M Hogan D Global, regional, and national estimates of levels of preterm birth in 2014: a systematic review and modelling analysis. Lancet Glob Health. (2019) 7(1):e37–46. 10.1016/S2214-109X(18)30451-0 30389451 PMC6293055

[47] Xu Y Liu H Hao D Taggart M Zheng D. Uterus modeling from cell to organ level: towards better understanding of physiological basis of uterine activity. IEEE Rev Biomed Eng. (2022) 15:341–53. 10.1109/RBME.2020.3023535 32915747

[48] Li X Kruger JA Nash MP Nielsen PMF. Modeling childbirth: elucidating the mechanisms of labor. Wiley Interdiscip Rev Syst Biol Med. (2010) 2(4):460–70. 10.1002/wsbm.65 20836041

[49] Means SA Roesler MW Garrett AS Cheng L Clark AR. Steady-state approximations for hodgkin-huxley cell models: reduction of order for uterine smooth muscle cell model. PLoS Comput Biol. (2023) 19(8):e1011359. 10.1371/journal.pcbi.1011359 37647265 PMC10468033

[50] Garrett AS Means SA Roesler MW Miller KJW Cheng LK Clark AR. Modeling and experimental approaches for elucidating multi-scale uterine smooth muscle electro- and mechano-physiology: a review. Front Physiol. (2022) 13:1017649. 10.3389/fphys.2022.1017649 36277190 PMC9585314

[51] Wu W Wang H Zhao P Talcott M Lai S McKinstry RC Noninvasive high-resolution electromyometrial imaging of uterine contractions in a translational sheep model. Sci Transl Med. (2019) 11(483):eaau1428. 10.1126/scitranslmed.aau1428 30867320

[52] Wang H Wen Z Wu W Sun Z Kisrieva-Ware Z Lin Y Noninvasive electromyometrial imaging of human uterine maturation during term labor. Nat Commun. (2023) 14(1):1198. 10.1038/s41467-023-36440-0 36918533 PMC10015052

[53] Wang S Anderson K Pizzella S Xu H Wen Z Lin Y Noninvasive electrophysiological imaging identifies 4D uterine peristalsis patterns in subjects with normal menstrual cycles and patients with endometriosis. Res Sq [Preprint]. (2023):rs.3.rs-2432192. 10.21203/rs.3.rs-2432192/v1

[54] Katsoulakis E Wang Q Wu H Shahriyari L Fletcher R Liu J Digital twins for health: a scoping review. NPJ Digit Med. (2024) 7(1):77. 10.1038/s41746-024-01073-0 38519626 PMC10960047

[55] Bai J Lu Y Wang H Zhao J. How synergy between mechanistic and statistical models is impacting research in atrial fibrillation. Front Physiol. (2022) 13:957604. 10.3389/fphys.2022.957604 36111152 PMC9468674

[56] Venkatesh KP Raza MM Kvedar JC. Health digital twins as tools for precision medicine: considerations for computation, implementation, and regulation. NPJ Digit Med. (2022) 5(1):150. 10.1038/s41746-022-00694-7 36138125 PMC9500019

[57] Coorey G Figtree GA Fletcher DF Snelson VJ Vernon ST Winlaw D The health digital twin to tackle cardiovascular disease-a review of an emerging interdisciplinary field. NPJ Digit Med. (2022) 5(1):126. 10.1038/s41746-022-00640-7 36028526 PMC9418270

[58] Stahlberg EA Abdel-Rahman M Aguilar B Asadpoure A Beckman RA Borkon LL Exploring approaches for predictive cancer patient digital twins: opportunities for collaboration and innovation. Front Digit Health. (2022) 4:1007784. 10.3389/fdgth.2022.1007784 36274654 PMC9586248

[59] Cappon G Vettoretti M Sparacino G Favero SD Facchinetti A. ReplayBG: a digital twin-based methodology to identify a personalized model from type 1 diabetes data and simulate glucose concentrations to assess alternative therapies. IEEE Trans Biomed Eng. (2023) 70(11):3227–38. 10.1109/TBME.2023.3286856 37368794

[60] Wickramasinghe N Jayaraman PP Forkan ARM Ulapane N Kaul R Vaughan S A vision for leveraging the concept of digital twins to support the provision of personalized cancer care. IEEE Internet Comput. (2021) 26(5):17–24. 10.1109/MIC.2021.3065381

[61] Davidson L Boland MR. Towards deep phenotyping pregnancy: a systematic review on artificial intelligence and machine learning methods to improve pregnancy outcomes. Brief Bioinform. (2021) 22(5):bbaa369. 10.1093/bib/bbaa369 33406530 PMC8424395

[62] Drukker L Noble JA Papageorghiou AT. Introduction to artificial intelligence in ultrasound imaging in obstetrics and gynecology. Ultrasound Obstet Gynecol. (2020) 56(4):498–505. 10.1002/uog.22122 32530098 PMC7702141

[63] Sarno L Neola D Carbone L Saccone G Carlea A Miceli M Use of artificial intelligence in obstetrics: not quite ready for prime time. Am J Obstet Gynecol MFM. (2023) 5(2):100792. 10.1016/j.ajogmf.2022.100792 36356939

[64] Du Y McNestry C Wei L Antoniadi AM McAuliffe FM Mooney C. Machine learning-based clinical decision support systems for pregnancy care: a systematic review. Int J Med Inform. (2023) 173:105040. 10.1016/j.ijmedinf.2023.105040 36907027

